# Patient experiences and the association with organizational factors in general practice: results from the Norwegian part of the international, multi-centre, cross-sectional QUALICOPC study

**DOI:** 10.1186/s12913-016-1684-z

**Published:** 2016-08-24

**Authors:** Torunn Bjerve Eide, Jørund Straand, Hasse Melbye, Guri Rortveit, Irene Hetlevik, Elin Olaug Rosvold

**Affiliations:** 1 Department of General Practice, Institute for Health and Society, University of Oslo, Oslo, Norway; 2 General Practice Research Unit, UiT The Arctic University of Norway, Tromsø, Norway; 3 Research Unit for General Practice, Uni Research Health, Bergen, Norway; 4 Department of Global Public Health and Primary Care, University of Bergen, Bergen, Norway; 5 General Practice Research Unit, Department of Public Health and General Practice, Norwegian University of Science and Technology, Trondheim, Norway

**Keywords:** Primary health care, General practice, Patient satisfaction, Physician-patient relations, QUALICOPC, Norway, Health-services administration

## Abstract

**Background:**

General practitioners (GPs) constitute a vital part of a strong primary health care system. We need further knowledge concerning factors that may affect the patients’ experiences in their meetings with the GPs. We investigated to what degree organizational factors and GP characteristics are associated with patients’ communicative experiences in a consultation.

**Methods:**

We used data from the Norwegian part of the international, multi-center study Quality and Costs of Primary Care in Europe (QUALICOPC). We included 198 Norwegian GPs and 1529 patients. The patients completed a survey concerning experiences in a consultation with a GP on the inclusion day. The GPs completed a survey regarding organizational aspects of their own practice. Main outcome measures were seven statements concerning how the patients experienced the communication with the GP during the consultation. A generalized estimating equation logistic regression model was used to identify variations in patient experiences associated with characteristics of the GPs and their practices.

**Results:**

The patients reported overall positive experiences with their GP consultations. Patients who consulted a GP with a short patient list were less likely than patients who consulted a GP with a medium sized list to regard the GP as polite (Odds Ratio (OR) 0.2; 95 % CI 0.1–0.7), to report that the GP asked questions about their health problems (OR 0.6; 0.4–1.0) or that the GP used sufficient time (OR 0.5; CI 0.3–0.9). Patients who consulted a GP with a long patient list compared to patients who consulted a GP with a medium sized list were less likely to feel that they could cope better after the GP visit (OR 0.5; 0.3–0.9) and more likely to feel that the GP hardly looked at them while talking (OR 1.8; 1.0–3.0). No associations with patient experiences were found with the average duration of the consultations, whether the GP worked in a fee-for-service model or whether the GP was the patient’s regular doctor.

**Conclusions:**

Norwegian patients report predominantly positive experiences when consulting a GP. Positive communication experiences are most likely to be reported when the GP has a medium sized patient list.

## Background

Primary health care is increasingly acknowledged as the linchpin of a strong health care system [1, 2]. Most European health authorities have a common vision of a strong primary health care system, but there is substantial inter-country variation of the frame-work provided for the GPs’ work. Reforms are frequently discussed or being implemented, and knowledge concerning aspects that may affect the quality of primary health care provision is of value to political decision-makers. Three main dimensions of primary care have been identified: *Structure* (governance, economic conditions and workforce development), *process* (access, continuity of care, coordination of care and comprehensiveness of care), and *outcome* (quality of care, efficiency of care, equity in health) [3]. Patients’ perceived satisfaction with the medical help they receive from their GP will to a large extent be coloured by the process-aspects, while it is more difficult for lay people to evaluate the medical quality and appropriateness of received care.

Patient satisfaction has been commonly used as an indicator of the quality of primary health care systems and individual health suppliers in different contexts [4, 5]. The concept of quality as applied in health services research is, however, often unclear, and the definitions vary [6]. Over the recent years the tendency has been to survey patients’ actual experiences instead of evaluating their more general satisfaction with health care services [7, 8]. We have scarce information on whether the organisational aspects of primary care may affect the patients’ experiences.

The main aspects of consultations with a GP, as judged by patients, have been reported to be the interaction with the doctor and the outcome of the consultation [9]. In addition, information, continuity of care, and available time with the doctor were considered important factors. In a recent Norwegian study, there was an association between the patients’ satisfaction with the access to care and the GPs’ service production, whereas no associations were found with time spent in consultation or whether the patients perceived that the GP took their medical problem seriously [10].

The frame-work of primary care varies throughout Europe. In Norway most GPs are self-employed, and as such have substantial freedom in terms of how they organize their practices [11, 12]. There are considerable differences when it comes to the size of the GPs’ patient lists, the number of colleagues with shared facilities, whether they employ nurses or health secretaries, how many days per week and hours per day they choose to be in office, which medical procedures they carry out, whether they offer home visits and to what extent they are reachable for the patients by phone, SMS or e-mail. Through the annual Commonwealth Fund International Health Policy Survey, we have information both regarding GPs evaluation of their own practices and their interaction with the health care systems [13], and about patients’ experiences with the primary care system [14, 15]. There are, however, few studies that permit analyses based on linked information between individual patients and their regular GP, and we therefore have little knowledge regarding how organizational aspects in the GP’s practice affect the patients’ experience. With the present study, we wish to investigate this potential association. We analyze Norwegian data with the aim to identify how the patients’ experiences vary with characteristics of the corresponding GPs and the organisational factors of their practices.

## Methods

The QUALICOPC (Quality and Costs of Primary Care in Europe) study is a multi-centre study that comprises 34 countries [16]. A set of four questionnaires was developed by the QUALICOPC Partner Consortium, led by the Netherlands Institute for Health Services Research (NIVEL). The rational of the construction of the questionnaires and the full version of their content has been published elsewhere [17]. The questionnaires were translated into the languages of the participating countries by a “forth and back” translation procedure, and a few of the questions were adjusted to fit the different national settings. The survey set consisted of: 1) A GP questionnaire concerning organisational aspects of the GP’s practice, the health problems and procedures handled in the practice and the range of medical equipment available for the GP. 2) A patient questionnaire concerning experiences with one specific GP consultation and with this GP’s practice, and also concerning which health problems the patients expected the GP to be of help with. 3) A patient questionnaire concerning how the patients valued the different aspects of primary care. 4) A fieldworker questionnaire concerning the practice facilities. In each participating medical practice, fieldworkers consecutively invited ten patients ≥18 years who had a face-to-face consultation with the participating GP on a randomly selected day. The patients’ surveys were completed in the GPs waiting room on the day of the consultation. Per participating GP, nine patients answered the patient experiences survey, one patient answered the patient values survey, and one fieldworker survey per GP was completed. Fieldworkers in Norway were either study coordinators, students or health secretaries working in the practice. The fieldworkers and the participating GPs each received a gift voucher of approximately 45 euro. The patients did not receive payment for participation. All GP and patient surveys were answered anonymously. A unique identification number linked GP responses to the responses of his/her patients and the fieldworker survey.

### Sample

The study is based on data from the Norwegian part of the QUALICOPC study. Data collection took place from November 2012 to April 2013. The four Norwegian General Practice Research Units at the Universities of Oslo, Trondheim and Tromsø and the research institute Uni Research Health in Bergen were all involved in recruiting doctors and patients to the study, thus ensuring that we received information from the entire country. GPs were contacted via formal and informal GP networks, and those who were willing to participate were sent a survey set or received a visit from a fieldworker. The Norwegian material consists of information from 198 GPs and 1704 patients. In total, 1529 patient completed the experience form and 175 completed the values form.

### Measures

The present study uses data from the patient experiences and the GP questionnaires. Table 1 presents an overview of all variables included in our analyses. The following independent variables described the organisational features of the GP’s practice: the size of the patient list, the average consultation time as judged by the individual GP, whether the GP had a fixed salary or a fee for service system, and the geographical location of the practice. We identified seven outcome variables that gave information on how the patients experienced their visit at the doctor’s office and, in particular, the communication with the doctor (Table 1). Continuous variables were transformed into categorical data as indicated in Table 1.

**Table 1:** Items from the QUALICOPC questionnaires included as variables in this study

Variables	Response alternatives
Information from the patients	
Gender	Male/female
Age	Years (<30, 30–65, >65)^a^
Did you see your regular doctor today?	Yes/No
The doctor was polite	Yes/No
The doctor listened carefully to me	Yes/No
The doctor asked questions about my health problem	Yes/No
The doctor took sufficient time in today’s consultation	Yes/No
The doctor hardly looked at me when we talked	Yes/No
I couldn’t really understand what the doctor was trying to explain	Yes/No
After this visit, I can cope better with my health problem/illness	Yes/No^b^
Information from the doctor	
Gender	Male/female
Age	Years (≤35, 36–59, ≥ 60)^a^
Geographical location of practice	1. Big city /Suburbs/ Small town; grouped as Urban 2. Mixed urban–rural / Rural; grouped as Rural
Size of patient list	Number of patients (≤900, 901–1300, >1300)^a^
Form of employment	Fixed salary / Fee for service
Duration of an average consultation (as assessed by the GP)	Minutes (≤17 min, >17 min)^a^

^a^The age of patients and doctors, the size of patient lists and the duration of consultations were all continuous variables divided into groups before analysis. Groups were defined according to the distribution of the material (see Tables 2 and 3)

^b^401 patients answered “I don’t know”. These were recoded into missing

### Statistical analysis

Due to the hierarchical structure of the data, we used a generalized estimating equation (GEE) logistic regression model. This modelling technique helped to account for the variability in patients’ experiences between the GPs and to establish any variation at the GP level. The significance level was set to *p* < 0.05.

All analyses were performed using SPSS statistics 22.

## Results

Tables 2 and 3 present demographic characteristics of the 1529 patients and 198 doctors. A majority of the patients (89.3 %) consulted with their regular doctor. Among the GPs, 39.1 % were female. The mean patient list size was 1093, with a tendency among the female GPs to have shorter lists than the male GPs (1049 versus 1123).

**Table 2:** Demographic data of participating patients (percentages in brackets)

	Total	Women	Men
Total	1529 (100)	916 (61.9)^a^	564 (38.1)^a^
Age^b^			
Range	18–93	18–91	18–93
Mean	48.7	46.2	52.5
Education^c^			
Primary school	194 (13.4)	118 (13.1)	75 (13.8)
High-school/college	591 (40.8)	355 (39.4)	236 (43.3)
Higher education	663 (45.8)	429 (47.6)	234 (42.9)
Visited their regular GP?^d^			
Yes	1321 (89.1)	796 (89.3)	482 (88.6)
Patients with a chronic condition^e^	764 (51.1)	445 (49.6)	289 (52.7)
Patient’s evaluation of own health^f^			
Very good	243 (16.2)	164 (18.3)	73 (13.2)
Good	741 (49.4)	428 (47.7)	289 (52.4)
Fair	382 (25.5)	222 (24.7)	149 (27.0)
Poor	133 (8.9)	84 (9.4)	41 (7.4)

Number of missing values: ^a^ 49, ^b^ 59, ^c^ 81, ^d^ 51, ^e^ 34, ^f^ 30

**Table 3:** Demographic data of participating GPs (percentages in brackets)

	Total	Female	Male
Total	198 (100)	77 (39.1)^a^	120 (60.9)^a^
Age			
Range	28–69	28–68	28–69
Mean	45.7	43.4	47
Born in Norway^b^	160 (81.6)	65 (84.4)	94 (79.7)
Geographical location of practice^c^			
Big inner city	66 (33.8)	29 (38.7)	36 (30.3)
Suburbs	27 (13.8)	12 (16.0)	15 (12.6)
Small town	44 (22.6)	14 (18.7)	30 (25.2)
Mixed urban–rural	31 (15.9)	7 (9.3)	24 (20.2)
Rural	27 (13.8)	13 (17.3)	14 (11.8)
Size of patient list^a^			
Range	250–1800	400–1500	250–1800
Mean	1093.4	1048.9	1122.6
Form of employment			
Fee for service	181 (91.4)	70 (90.9)	110 (91.7)
Fixed salary	17 (8.6)	7 (9.1)	10 (8.3)
Duration of average consultation as assessed by GP (minutes)			
Range	10–30	15–25	10–30
Mean	18.6	19.1	18.3

Number of missing values: ^a^ 1, ^b^ 2, ^c^ 3

Patients’ reports from their consultation with the GP were generally positive. A great majority of the patients reported that the GP was polite (97.9 %), listened carefully (97.1 %) and took sufficient time (91.1 %) (Table 4). Most patients (88 %) also reported that they could cope better with their health problems after the visit. On the other hand, few patients experienced that the doctor hardly looked at them (7.4 %) or that they could not understand what the doctor was trying to explain (8.0 %). Table 4 presents the number and percentage of patients giving a positive response to the statements in Table 1 for each of the subgroups of the GPs.

**Table 4:** Percentages of patients that answered yes to each question

	GP was polite^a^	GP listened carefully^a^	GP hardly looked at me when we talked^a^	GP asked questions about my health problem^a^	I couldn’t really understand what the GP was trying to explain^a^	GP took sufficient time^a^	After this visit, I can cope better with my health problem^b^
Total (*n* = 1529)	97.9	97.1	7.4	90.3	8.0	91.1	87.9
Patient’s gender^c^							
Male (545)	98.7	97.2	7.2	89.7	7.5	93.2	89.0
Female (890)	98.0	97.3	7.5	91.3	8.1	90.1	87.7
Patient’s age^d^							
< 30 (219)	99.1	97.7	7.8	94.5	9.1	93.6	84.2
30–65 (928)	98.3	97.5	6.4	90.9	6.6	90.5	86.9
> 65 (278)	97.5	95.7	9.7	87.1	10.8	92.1	97.3
Did you see your regular doctor?^e^							
Yes (1297)	98.1	97.5	7.5	90.9	7.6	92.0	88.8
No (156)	97.4	94.2	7.7	86.5	9.6	87.8	77.7
GP’s gender^f^							
Male (868)	98.0	97.7	7.6	90.4	7.6	91.5	88.5
Female (546)	97.8	96.3	6.6	90.7	8.6	91.6	88.1
GP’s age^g^							
< =35 (203)	98.0	99.0	8.4	91.1	9.9	91.6	87.8
36–59 (1085)	98.0	96.9	7.2	91.2	8.1	91.4	88.1
> =60 (133)	97.7	97.0	5.3	84.2	3.8	92.5	92.0
Geographical location^h^							
Urban (1042)	98.1	97.5	6.7	91.4	7.2	91.7	88.2
Rural (366)	97.5	96.2	8.7	88.3	10.4	91.0	88.7
List size^i^							
>1300 (367)	98.6	98.0	9.6	91.8	9.0	90.1	85.5
900–1300 (784)	98.4	96.7	5.9	91.1	7.3	93.2	90.8
< 900 (327)	96.1	97.4	7.7	88.1	8.7	89.4	85.9
Employment of GP^j^							
Fixed salary (81)	97.5	97.5	3.7	90.1	8.6	93.8	85.4
Fee for service (1340)	98.0	97.2	7.4	90.6	7.9	91.4	88.5
Average duration of consultation^k^							
≤ 17 min (498)	97.9	97.7	7.4	89.7	7.8	89.1	87.1
> 17 min (967)	98.2	97.1	7.1	91.2	8.1	92.9	88.9

Number of missing values: ^a^49, ^b^518 (see Table 1), ^c^94, ^d^104, ^e^76, ^f^115, ^g^108, ^h^121, ^i^102, ^j^ 108, ^k^64

The left column indicates subgroups of patients according to characteristics of the patients or the GP they attended

Table 5 presents the results of the multivariate GEE logistic regression analyses. When analysing the impact of list size, we defined the patients visiting GPs with a medium sized list (901–1300 patients) as the reference group. Patients visiting a GP with a shorter patient list were less likely to respond positively to the statements “The doctor was polite” (OR 0.2; CI 0.1–0.7), “The doctor asked questions about my health problem” (OR 0.6; CI 0.4–1.0) and “The doctor took sufficient time” (OR 0.5; CI 0.3–0.9). Patients visiting a GP with a longer patient list were less likely to answer yes to the statement “After this visit, I can cope better with my health problem/illness” (OR 0.5; CI 0.3–0.9). When using patients that visited GPs with smaller lists as the reference group, no additional significant differences were found.

**Table 5:** Associations between patients’ experiences and characteristics of the patients, GPs and the GP practices

	GP was polite	GP listened carefully	GP hardly looked at me when we talked	GP asked questions about my health problem	I couldn’t really understand what the GP was trying to explain	GP took sufficient time	After the visit, I can cope better with my health problem
	OR (95 % CI)	OR (95 % CI)	OR (95 % CI)	OR (95 % CI)	OR (95 % CI)	OR (95 % CI)	OR (95 % CI)
Patient’s gender							
Male (ref)							
Female	0.5 (0.2–1.4)	1.0 (0.5–2.2)	1.0 (0.7–1.6)	1.0 (0.7–1.5)	1.2 (0.8–1.9)	0.7 (0.4–1.1)	1.0 (0.6–1.6)
Patient’s age							
< 30	#	1.7 (0.5–6.3)	1.3 (0.7–2.3)	**2.3 (1.1–4.6)** ^**^	1.4 (0.8–2.4)	1.8 (1.0–3.5)	0.8 (0.5–1.5)
30–65 (ref)							
> 65	#	0.6 (0.3–1.2)	**1.7 (1.0–2.9)** ^**^	0.7 (0.4–1.1)	**2.0 (1.1–3.4)** ^**^	1.1 (0.6–2.1)	**4.7 (1.8–12.3)** ^*^
Regular doctor?							
No (ref)							
Yes	0.9 (0.2–3.8)	2.7 (1.0–6.9)	0.8 (0.4–1.6)	1.7 (0.9–3.0)	0.6 (0.4–1.2)	1.5 (0.8–2.9)	1.5 (0.7–3.1)
GP’s gender							
Male (ref)							
Female	0.5 (0.2–1.5)	0.5 (0.3–1.1)	0.9 (0.6–1.4)	0.8 (0.5–1.2)	1.1 (0.8–1.7)	0.8 (0.5–1.3)	0.9 (0.6–1.6)
GP’s age							
≤ 35	1.2 (0.3–5.6)	2.9 (0.7–11.4)	1.2 (0.6–2.2)	1.1 (0.6–2.0)	1.4 (0.8–2.4)	1.0 (0.6–1.8)	1.1 (0.6–2.0)
36–59 (ref)							
≥ 60	0.6 (0.1–2.2)	1.1 (0.4–3.6)	0.7 (0.2–2.1)	**0.5 (0.3–0.8)** ^*^	0.4 (0.2–1.2)	1.1 (0.6–2.1)	1.4 (0.6–3.3)
Geographical location							
Urban (ref)							
Rural	0.9 (0.3–2.4)	0.6 (0.3–1.3)	1.6 (1.0–2.5)	0.8 (0.5–1.3)	**1.8 (1.2–3.0)** ^**^	0.9 (0.5–1.4)	0.9 (0.5–1.7)
Size of patient list							
> 1300	0.9 (0.2–4.2)	1.5 (0.5–4.4)	1.8 (1.0–3.0)**	1.0 (0.6–1.7)	1.4 (0.7–2.5)	0.7 (0.4–1.3)	**0.5 (0.3–0.9)** ^**^
900–1300 (ref)							
< 900	0.2 (0.1–0.7)*	1.3 (0.5–3.4)	1.2 (0.7–2.1)	**0.6 (0.4–1.0)** ^**^	1.0 (0.6–1.6)	**0.5 (0.3–0.9)** ^**^	0.7 (0.3–1.4)
Employment							
Fixed salary (ref)							
Fee for service	0.4 (0.0–3.5)	0.7 (0.1–4.1)	2.7 (0.9–7.9)	0.8 (0.3–1.9)	1.1 (0.5–2.4)	0.6 (0.2–1.5)	1.1 (0.4–3.4)
Average duration of consultation							
≤ 17 min, ref							
> 18 min	1.2 (0.3–4.2)	0.8 (0.3–2.4)	1.0 (0.7–1.7)	1.1 (0.7–1.6)	1.0 (0.6–1.7)	1.5 (0.9–2.5)	1.1 (0.6–1.8)

*p < 0.005, **p < 0.05, # Too few respondents in one of the categories, ref = reference group

The table shows the results of multivariate cluster analyses (generalized estimating equations). For the dependent variables in the top row, odds ratio indicates the probability of the answer yes. The left column indicates subgroups of patients according to characteristics of the patients or of the GP they attended. Bold figures indicate statistically significant associations

Patients visiting a GP in a rural area were more likely to answer yes to the statement “I couldn’t really understand what the doctor was trying to explain” (OR 1.8; CI 1.2–3.0) compared to patients visiting doctors in an urban area.

When analysing the impact of the doctors’ age, the middle age group (36–59 years) was defined as reference. Patients visiting a GP aged 60 years or more were less likely to answer yes to the statement “The doctor asked questions about my health problem” (OR 0.5; CI 0,3–0,8). This was also true when compared to patients visiting GPs in the youngest age group (OR 0.4; CI 0.2–0.9). This was the only significant association found with the GPs’ age.

When analysing the impact of the patients’ age, we also defined the middle age group (30–65 years) as the reference. Patients less than 30 years old were more likely to answer yes to whether the doctor asked questions about their health problems (OR 2.3; CI 1.1–4.6). Patients above 65 years were more likely than the middle aged patients to answer yes to the statements “The doctor hardly looked at me when we talked” (OR 1,7; CI 1.0–2.9), and “After this visit, I can cope better with my health problem/illness” (OR 4.7; CI 1.8–12.3). When using the youngest age group as reference, additional differences were identified: Both the middle age group (OR 0.4; CI 0.2–0.9) and the oldest age group (OR 0.3; CI 0.1–0.6) were less likely to state that the GP asked additional questions. The oldest patients were more likely to feel that they could cope better after the visit to the GP than the youngest patients (OR 5.7; CI 1.9–16.5). The oldest age group was more likely to answer yes to the statement “I couldn’t really understand what the doctor was trying to explain” (OR 2.0; CI 1.1–3.4) compared to the middle age group, but no difference was found when compared to the youngest age group.

We found no associations between the patients’ experiences and the GPs’ form of employment, the average duration of consultation estimated by the GPs or whether the GP was the patient’s regular doctor or not.

## Discussion

The patients in our material reported an overall positive experience with their GP consultations. The patients’ experiences were to a certain extent influenced by the size of the GP’s patient list, the geographical location of the practice and the GP’s age. Among these effects, the list size stands out as the most influential, and both short and long patient lists were associated with a more negative patient experience. We also found that the patients’ age was of significance as to how they perceived their visit to the GP. The older patients were more likely to experience that the doctor did not look at them while talking, and they found it more difficult to understand what the GP tried to explain. Nevertheless, they were also more likely to feel that they could cope better with their health problems after the visit to the GP.

### Interpretation of results and comparisons with previous studies

We found an association between the size of the GPs patient lists and the patients’ experiences. Intuitively, and based on previous reports [18–20], one might expect that GPs with shorter patient lists will have more time per patient, rendering the patient with an experience of a doctor that takes enough time and makes sure to ask supplementary questions to the patient’s story [19]. This was not found in our study. Patients who saw GPs with shorter patient lists reported relatively more negative experiences with regard to time spent, the doctor’s politeness and whether the GP asked for more information. Shorter patient lists may be due to less time in the practice, lower work capacity for the individual doctor, or not having achieved the warranted number of patients on the list. The last situation may occur when the practice is newly established or because the doctor simply is not popular among patients. Less time present in the practice may be due to additional employments. Private reasons such as having young children or personal illness may be reasons for reduced capacity of the individual doctor. We did not have data to further explore these different reasons for the associations found.

On the other hand, patients who attended GPs with the largest patient lists were more likely to report that the doctor hardly looked at them while talking and less likely to feel that they could cope better with their health problems after the visit to the GP. It is possible that the busy doctors with the largest patient lists devote less time to making sure that their intended message has been received by the patients, and with a higher patient turnover there may be less awareness towards the patients’ need to ask clarifying questions.

A Dutch study concluded that the optimum practice size with regard to the physicians’ workload was found in the largest practices, but they did not investigate the effects on patients’ experiences [21]. In a recent Swiss study, a higher satisfaction rate was found in smaller practices measured by the number of GPs working in the practice, but the study did not explore the effect of the number of patients in each practice [22]. Studies on practice size are not always directly comparable between countries, as systems differ with regard to how the patient population of the individual GP is defined. A possible interpretation of our findings with regard to the size of patient lists is that, from the patients’ point of view, a GP should have a list of a certain size to ensure satisfactory service, but that there seems to be an upper limit for the list size to allow for adequate patient care. Further research concerning GPs’ reasons for having short and long patient lists will be of interest to contribute to the understanding of our findings.

Patients who consulted a GP in a rural setting were more likely to report that they had problems understanding what the GP was trying to explain. This could be due to language problems, as GPs with an immigrant background more often than other GPs work in rural areas of Norway [23].

A recent paper based on the international results from the QUALICOPC study investigated the patients’ evaluation of the importance of different aspects of the contact with the GP [24]. The Norwegian patients valued involvement and communication highly, underlining the importance of identifying factors that may affect the patients’ experiences in these areas. Overall, we did not find that organizational factors had a large impact on how patients experienced their visit to the GP when focusing on the communicative interaction between doctor and patient. A possible reason for this is that Norwegian inhabitants may freely choose their regular GP provided that there is sufficient availability of GPs in the relevant geographical region. It is probable that people choose a doctor whose communicative style fits their own preferences. Patients should not be seen as a homogenous group that all expect and prefer the same qualities in a doctor. The diversity of Norwegian GPs may therefore be regarded as a positive quality that gives the inhabitants the possibility of choosing a personal doctor who provides services in a manner preferable to the individual.

### Strengths and limitations

The recruitment procedure for the participating GPs was suboptimal in terms of obtaining a random selection. The GP population in the study is a convenience sample recruited through formal and informal networks of the four participating research units, and is therefore not necessarily a fully representative sample of Norwegian GPs. However, the GPs were recruited from various geographical regions throughout the country. Some of the GPs have university affiliations and may therefore be more positive than most GPs towards participations in research projects. In 2013, 38.6 % of Norwegian GPs were female [12], in our material 29.1 % of the GPs were female. The average age of Norwegian GPs in 2013 was 48.1 years, whereas the GPs in our material were slightly younger with a mean of 45.7 years. The mean patient list size per GP in Norway in 2013 was 1150 patients [12], whereas in our material it was somewhat smaller with a difference of 57 patients. In 2013, 4.7 % of the Norwegian GPs were on regular salaries [12], compared to 8.6 % in our material. GPs on regular salaries might be more likely to take part in research projects during their regular work hours, as this will not affect their income. The differences are small, and we therefore assume that our material is representative of the Norwegian GP population.

As our data origins from a larger, international study, it was not possible to fully customize the questions to Norwegian conditions or to the specific needs of the present national study. Information about how many days per week the GPs work in their practices and more detailed information about the geographical location would have been of value to our study.

The strength of our study lies in the size of the material and the possibility to link information from the patients with detailed information from the actual GP they attended. The data were obtained from all over the country and are representative of the Norwegian GP population. The patients answered the questions in the GPs’ waiting room, thus minimizing the potential for recall bias. Since the organisation of Norwegian general practice is rather diverse, we believe that our findings are of relevance even when evaluating primary care systems in other countries.

## Conclusion

Norwegian patients report predominantly positive experiences when visiting a general practitioner.

Both short and long patient lists were associated with various negative patient experiences in the consultation with the GP. A rural location of the GP practice was negatively associated with the communicative experience of the patients.

Our study suggests that from the patients’ point of view, it is preferable for GPs to have a medium size patient list to allow for a positive communicative experience in the consultation.

## Abbreviations

GEE: General estimating equations
GP: General practitioner
NIVEL: Netherlands Institute for Health Services Research
QUALICOPC: Quality and Costs of Primary Care in Europe

## References

[1] Starfield B, Shi L, Macinko J (2005). Contribution of primary care to health systems and health. Milbank Q.

[2] Kringos D, Boerma W, Bourgueil Y, Cartier T, Dedeu T, Hasvold T (2013). The strength of primary care in Europe: an international comparative study. Br J Gen Pract.

[3] Kringos DS, Boerma WG, Hutchinson A, van der Zee J, Groenewegen PP (2010). The breadth of primary care: a systematic literature review of its core dimensions. BMC Health Serv Res.

[4] Sixma HJ, Spreeuwenberg PM, van der Pasch MA (1998). Patient satisfaction with the general practitioner: a two-level analysis. Med Care.

[5] Sanchez-Piedra CA, Prado-Galbarro FJ, Garcia-Perez S, Santamera AS (2014). Factors associated with patient satisfaction with primary care in Europe: results from the EUprimecare project. Qual Prim Care.

[6] Grepperud S (2009). Quality in health care--what does it mean actually?. Tidsskr Nor Laegeforen.

[7] Salisbury C, Wallace M, Montgomery AA (2010). Patients’ experience and satisfaction in primary care: secondary analysis using multilevel modelling. BMJ.

[8] Sixma HJ, Kerssens JJ, Campen C, Peters L (1998). Quality of care from the patients’ perspective: from theoretical concept to a new measuring instrument. Health Expect.

[9] Steine S, Finset A, Laerum E (2000). What is the most important for the patient in the meeting with a general practitioner?. Tidsskr Nor Laegeforen.

[10] Grytten J, Carlsen F, Skau I (2009). Services production and patient satisfaction in primary care. Health Policy.

[11] The Norwegian Medical Association. http://legeforeningen.no/Emner/Andre-emner/Legestatistikk/Yrkesaktive-leger-i-Norge/Legeforeningens-fastlegestatistikk---artikkel/. Accessed 28 Oct 2015

[12] The Norwegian Directorate of Health. https://helsedirektoratet.no/statistikk-og-analyse/fastlegestatistikk. Accessed 6 May 2015

[13] Haugum M BØ, Iversen HH, Lindahl AK, Nylenna M. Commonwealth Fund survey among primary care physicians in 10 countries: Norwegian results in 2012 and development since 2009. In: Rapport fra Kunnskapssenteret. vol. 11–2012: The Norwegian Knowledge Centre for the Health Services; 2012.

[14] Holmboe O IH, Sjetne IS, Skudal K. Commonwealth Fund-undersøkelsen i 2011 blant utvalgte pasientgrupper: Resultater fra en komparativ undersøkelse i 11 land. In: Rapport fra Kunnskapssenteret. vol. 18–2011: The Norwegian Knowledge Centre for the Health Services; 2011.

[15] Sjetne IS SK, Haugum M, Bjertnæs ØA, Lindahl AK, Nylenna M. Commonwealth Funds undersøkelse i 2014 blant personer i aldersgruppe 55 år eller eldre: Resultater fra Norge og ti andre land. In: Rapport fra Kunnskapssenteret. vol. 21–2014: The Norwegian Knowledge Centre for the Health Services; 2014.

[16] Schafer WL, Boerma WG, Kringos DS, De Maeseneer J, Gress S, Heinemann S (2011). QUALICOPC, a multi-country study evaluating quality, costs and equity in primary care. BMC Fam Pract.

[17] Schafer WL, Boerma WG, Kringos DS, De Ryck E, Gress S, Heinemann S (2013). Measures of quality, costs and equity in primary health care instruments developed to analyse and compare primary care in 35 countries. Qual Prim Care.

[18] Campbell JL (1996). The reported availability of general practitioners and the influence of practice list size. Br J Gen Pract.

[19] Hasvold T (2000). Size of the patient list and quality of the general practice. Tidsskr Nor Laegeforen.

[20] van den Hombergh P, Kunzi B, Elwyn G, van Doremalen J, Akkermans R, Grol R (2009). High workload and job stress are associated with lower practice performance in general practice: an observational study in 239 general practices in the Netherlands. BMC Health Serv Res.

[21] Wensing M, van den Hombergh P, Akkermans R, van Doremalen J, Grol R (2006). Physician workload in primary care: what is the optimal size of practices? A cross-sectional study. Health Policy.

[22] Sebo P, Herrmann FR, Haller DM (2015). How do GPs in Switzerland perceive their patients’ satisfaction and expectations? An observational study. BMJ Open.

[23] Diaz E, Raza A, Sandvik H, Hjorleifsson S (2014). Immigrant and native regular general practitioners in Norway. A comparative registry-based observational study. Eur J Gen Pract.

[24] Schäfer WLABW, Murante AM, Sixma HJM, Schellevis FG, Groenewegen PP (2015). Assessing the potential for improvement of primary care in 34 countries: a cross-sectional survey. Bull World Health Organ.

